# Flaxseed Oil and Heated Flaxseed Supplements Have Different Effects on Lipid Deposition and Ileal Microbiota in Albas Cashmere Goats

**DOI:** 10.3390/ani11030790

**Published:** 2021-03-12

**Authors:** Shulin Liu, Xue Wang, Yinhao Li, Binlin Shi, Xiaoyu Guo, Yanli Zhao, Sumei Yan

**Affiliations:** Inner Mongolia Key Laboratory of Animal Nutrition and Feed Science, College of Animal Science, Inner Mongolia Agricultural University, Huhhot 010018, China; liushulin0723@163.com (S.L.); wangxue199204@163.com (X.W.); yinhaoli2019@126.com (Y.L.); shibinlin@yeah.net (B.S.); gxy_2594@163.com (X.G.); ylzhao2010@163.com (Y.Z.)

**Keywords:** flaxseed, flaxseed oil, lipid deposition, ileal microbiota, cashmere goats

## Abstract

**Simple Summary:**

With the grassland desertification intensified, pasture carrying capacity decreased, and grass seasonal changed, stall-feeding fattening has become an effective means to protect the natural environment. The stall-feeding fattening increased the cashmere goats’ weight but reduced the meat quality and increased the saturated fatty acids content in muscle and fat tissue of cashmere goats. Supplementing flaxseed and flaxseed oil rich-in linolenic acid (ALA) to diet to improve meat quality is an effective nutritional regulation means. Previous research results of our team showed that compared to diet supplemented with flaxseed oil, added flaxseed increased linoleic acid biohydrogenation by reducing the *Ruminobacter* relative abundance and increasing the relative abundance of *Prevotellaceae_UCG-001* and *Fretibacterium* in rumen, protected ALA away from biohydrogenation, and more n-3 polyunsaturated fatty acids entered the post-intestinal tract. Based on the previous research, this study explored whether the ALA flowing into the posterior intestine can reduce fat deposition and blood lipid by affecting intestinal microbiota. The results showed that adding flaxseed grain to diet decreased the growth performance, lipid deposition, and blood lipid content of goats by regulating the blood lipid-related enzyme activity, which positively associated with *[Eubacterium]_coprostanoligenes_group*, but negatively associated with *unclassified_f_Peptostreptococcaceae*, *Intestinibacter*, and *Ruminococcus_2*.

**Abstract:**

The present study investigated the effect of flaxseed grain or flaxseed oil on ileal microbiota and lipid deposition of cashmere goats. Sixty kid goats (average body weight 18.6 ± 0.1 kg) were allocated to three treatments, fed for 90 days, with control treatment: basal diet (CON, total-mixed ration), experimental treatment: basal diet with added flaxseed oil (LNO), experimental treatment: basal diet with added heated flaxseed grain (HLS). The final body weight, body weight gain, the weight of kidney fat, omental fat, tail fat, and fat tissue, the activity of fatty acid synthetase, acetyl-coa carboxylase, and malic dehydrogenase, and the relative abundance (RA) of *unclassified_f_Peptostreptococcaceae* and *Intestinibacter* were remarkably higher in the LNO treatment than in the HLS treatment, but the *[Eubacterium]_coprostanoligenes_group* RA showed the opposite result. The content of triglyceride, cholesterol, and low-density lipoprotein cholesterol were significantly higher in the CON and LNO treatments than in the HLS treatment, while the hormone-sensitive lipase activity and the non-esterified fatty acid content showed the opposite result. In conclusion, the flaxseed grain is more efficient than flaxseed oil in ameliorating the blood lipid profiles and it is a potential product for decreasing the lipid deposition of cashmere goats.

## 1. Introduction

Albas white cashmere goat is a world famous local breed producing cashmere and meat, coming from the plateau region of Ordos, Inner Mongolian, China. In recent years, due to the limitation of natural grassland resources, degeneration of the grassland, and the increase of mutton market demand, the fattening pattern has changed from traditional pasture fattening to feedlot fattening, which can alleviate the pressure of grassland and increase the economic benefits. However, changes in fattening pattern mean the alteration of diet nutritional level, which can cause metabolic changes in livestock. For lambs fed an energy-restricted feeding sequence program to mimic the seasonal changes of the natural grasslands, the results indicated that sequentially restricting metabolizable energy intake resulted in a reduction of body weight, serum glucose (GLU) and triglyceride (TG) concentration [1]. Feeding high-energy diets increased serum GLU and cholesterol (CHO) content in ewes [2]. Chen et al. reported that the diet with low ratio of forage to concentrate (F:C = 50:50) had a higher concentration of serum high-density lipoprotein cholesterol (HDL-C) in Yak than the diet with high-forage (F:C = 70:30, F:C = 60:40) groups [3]. Previous research reported an increase of plasma total lipids, CHO, TG, phospholipids, and non-esterified fatty acid (NEFA) in ruminant, which lead to lipid infiltration of the liver, and this favors the appearance of ketoacidosis, to the detriment of the health and reproductive performance of the animal [4]. Therefore, the increasing studies ameliorating the lipid metabolism of ruminants in stall fattening are very necessary. Manipulating the dietary fat might be a feasible approach to regulate blood lipid metabolism, however, very little information on ruminants is available. Flaxseed contains 32% to 45% of its mass as oil, of which 51% to 55% is a-linolenic acid (ALA) and 15% to 18% is linoleic acid, and it includes the flax lignan complex [5]. The main components of flaxseed oil are saturated fatty acid (SFA, 9%), monounsaturated fatty acid (18%), and ALA (57%) [6]. ALA is the most important fatty acid in flaxseed oil and flaxseed, which are the most important functional phytochemicals in flaxseeds for human health [7], and it is the precursor to the synthesis of eicosapentaenoic acid (EPA) and docosahexaenoic acid (DHA). The research observed that the addition of flaxseed could reduce the serum CHO concentration of cows [8], and another study reported that dietary calcium ALA could significantly reduce serum CHO, TG, and low-density lipoprotein cholesterol (LDL-C) levels in high-fat-fed mice [9]. Our previous study showed that flaxseed oil and flaxseed grain supplemented in diet showed different effects on increasing the concentration of ALA, EPA, DHA, and n-3 polyunsaturated fatty acids (n-3PUFA) in plasma and tissues in Albas cashmere goats, and flaxseed grain was more efficient [10]. n-3 PUFAs reduced plasma TG by approximately 20–30% and decreased the substrate of oxidative LDL-C and remnant CHO taken up by macrophages [11,12]. These researches hinted that flaxseed and flaxseed oil rich in n-3 PUFA may regulate the blood lipid profiles and lipid deposition, and they probably have different influences, but limited data are available, and the mechanism is also unclear.

Gut microbiota may be involved in the regulation of serum lipid levels and lipid accumulation, because it plays important roles in host metabolism [13]. Wen et al. reported that the genus *Methanobrevibacter* and the species *Mucispirillum schaedleri* were strongly associated with fat deposition in gut of chickens [14]. The bacteria in cecum of pigs positively associating with LDL-C/HDL-C in serum belongs to Proteobacteria, which is associated with inflammation [15]. An increase of serum LDL-C, very LDL-C and lipids concentration by dietary butyrate glycerides in broilers, not only significantly increased the *Bifdobacterium* abundance but also boosted the species diversity, and the results suggested the potential contribution of intestinal bacteria to lipid metabolism/energy homeostasis in broilers [16]. The previous research results of our team showed that compared to diet supplemented with linseed oil, added flaxseed increased linoleic acid biohydrogenation by reducing the relative abundance of *Ruminobacter* and increasing the relative abundance of *Prevotellaceae_UCG-001* and *Fretibacterium* in rumen, protected ALA away from biohydrogenation, and lead to more n-3 PUFAs entering the post-intestinal tract [17]. The previous studies have shown that except for the rumen and large intestine, the ileum also serves as an indispensable fermentation site in goats [18,19]. Yan et al. reported that there is a larger number of cellulolytic bacteria in sheep ileum, particularly *Clostridium cluster IV* [20]. Therefore, we hypothesized that ALA-rich flaxseed oil and flaxseed grain supplements have different effects in ameliorating blood lipid profiles and lipid deposition in Albas cashmere goats, and the mechanism is probably involved in altering ileal microbial composition. The current study aimed to test whether supplementing flaxseed oil and flaxseed grain in diet has different regulation on lipid deposition and ileal microbiota community in Albas cashmere goats, and to explain the possible mechanism of flaxseed oil and flaxseed in ameliorating lipid deposition.

## 2. Materials and Methods

The experiment was conducted in the experimental farm of Inner Mongolia Agricultural University (Hohhot, China). All animal procedures were performed under the national standard Guidelines for Ethical Review of Animal Welfare (GB/T 35892-2018).

### 2.1. Experimental Design, Diet, and Feeding Management

A single-factor completely randomized design was used. Sixty 4-month-old castrated Albas cashmere male kid goats were selected from a farm, at Etuoke Town, Inner Mongolia, with an initial live weight of 18.6 ± 0.1 kg, and were randomly assigned to three treatments, with each treatment comprising 4 units of 5 goats. The control group (CON) was fed the basal diet with no supplementation. The experimental group was fed the flaxseed oil-supplemented diets (LNO) prepared by manually blending the oil thoroughly into the ground concentrate to ensure homogenous distribution of the oil in the ration. Another experimental group was fed the basal diet supplemented with heated flaxseed grain (HLS, the flaxseed contains about 36% oil and it was stir roasted at 120 °C for 10 min), which provided the same content of flaxseed oil as the LNO group. The nutrition levels of the diet could meet the needs of growing cashmere kids, according to the feeding standard of meat-producing sheep and goats (NY/T816, 2004 [21]; Table 1). The trial consisted of a 14-day adaptation period and a 90-day treatment period, including early (1 to 30 days), medium (31 to 60 days), and late (61 to 90 days) fattening stages. The diet was offered to goats twice daily at 08:30 am and 16:30 pm as total mixed ration (concentrate to forage ratio of 50:50) and the goats were given free access to drinking water. To estimate feed intake for five goats in each pen, refusals were collected and weighed 30 min before each feeding, at 08:00 am daily. All goats were weighed (before feeding in the morning) on day 0 (initial body weight, IBW) and days 90 (final body weight, FBW) of the measurement periods to determine changes in body weight (total body weight gain, BWG).

### 2.2. Sample Collection

At the end of the experiment, 2 goats from each experimental unit (8 goats per treatment) were randomly selected for slaughtering by exsanguination. Before slaughter, the goats were prevented from consuming feed for 24 h and from drinking for 2 h. Jugular blood (20 mL) was sampled into Vacutainer tubes after the goats were prevented from consuming feed for 12 h. Blood was centrifuged at 3000× *g* for 15 min and serum was harvested, and at last stored at −20 °C for lipid profiles and enzymatic activity analysis. Immediately after death, the ileal digesta were collected, flash-frozen by liquid nitrogen, and stored at −80 °C for microbial diversity analysis.

### 2.3. Chemical Analysis

The concentrations of TG, CHO, LDL-C, and HDL-C in serum were analyzed in an automatic biochemical analyzer (L-8900) using commercially available kits (Lepu Diagnostics Co., Ltd., Beijing, China). The concentrations of NEFA, GLU, and β-hydroxybutyric acid (BHBA) in serum were analyzed through commercially available kits (Nanjing Jiancheng Bioengineering Institute, Nanjing, Jiangsu, China). The quantity of acetyl-coa carboxylase (ACC, CK-E75273), fatty acid synthetase (FAS, CK-E75339), malic dehydrogenase (MDH, CK-E75029), lipoprotein lipase (LPL, CK-E75274), hormone-sensitive lipase (HSL, CK-E75402), and lipase (LPS, CK-E75263) were measured by enzyme-linked immunosorbent assay (ELISA) kits (QUANZHOU RUIXIN BIOTECHNOLOGY CO., LTD., Quanzhou, Fujian, China), and the experiments were performed strictly according to the manufacturer’s instructions. Meanwhile, the standard curves of enzymes were established. Briefly, 10 μL serum sample and 40 μL sample diluent were added to the coated plate, and 100 μL diluted enzyme-labeled antibody was added to the plate, incubated at 37 °C for 1 h, then washed five times with phosphate-buffered saline (PBS) (let stand for 1 min). 100 μL substrate Tetramethylbenzidine (TMB) was added and avoided light incubation at 37 °C for 15 min, the reaction was terminated with 50 μL 2 M H_2_SO_4_, and the optical density (OD) was measured at 450 nm.

### 2.4. 16S rRNA Gene Sequencing and Operational Taxonomic Units (OTUs) Picking

#### 2.4.1. DNA Extraction and Checking

Five ileal digesta samples from each treatment group were selected to extract the microbial DNA using the E.Z.N.A.^®^ soil DNA Kit (Omega Bio-tek, Norcross, GA, USA) according to the instruction manual. The final DNA concentration and OD (Optical Density) 260/280 values were determined by NanoDrop 2000 UV-vis (ultraviolet-visible) spectrophotometer (Thermo Scientific, Wilmington, NC, USA), and DNA quality was checked through 1% agarose gel electrophoresis.

#### 2.4.2. PCR (Polymerase Chain Reaction) Amplification and Checking

The amplified region is V3–V4 hypervariable regions of the bacteria 16S rRNA gene. The amplified primers are 338F (5′-ACCHOCTACGGGAGGCAGCAG-3′) and 806R (5′-GGACTACHVGGGTWCHOTAAT-3′), and are universal primers. The procedure of PCR reactions was as follows: 3 min of denaturation at 95 °C, 27 cycles of 30 s at 95 °C, 30 s for annealing at 55 °C, and 45 s for elongation at 72 °C, and a final extension at 72 °C for 10 min. The mixed volume of PCR reactions was 20 μL: 4 μL of 5× FastPfu Buffer, 2 μL of 2.5 mM dNTPs (Deoxynucleotide Triphosphates), 0.8 μL of each primer (5 μM), 0.4 μL of FastPfu Polymerase, and 10 ng of template DNA. The PCR products were extracted from a 2% agarose gel and further purified using the AxyPrep DNA Gel Extraction Kit (Axygen Biosciences, Union City, CA, USA) and quantified using QuantiFluor™-ST (Promega, Madison, WI, USA) according to the instruction manual.

#### 2.4.3. Illumina MiSeq Sequencing

Purified amplicons were pooled on an Illumina MiSeq PE300 instrument (Illumina, San Diego, CA, USA) which were equimolar and paired-end sequenced (2 × 300) according to the standard protocols by Majorbio Bio-Pharm Technology Co. Ltd. (Shanghai, China).

#### 2.4.4. Bioinformatics

Raw data was filtered and analyzed using QIIME (Quantitative Insights into Microbial Ecology, version 1.9.1) software, quality-filtered by Trimmomatic and merged by FLASH (Fast Length Adjustment of Short Reads). Low-quality reads were removed with the following criteria: (i) The reads were truncated at any site receiving an average quality score < 20 over a 50 bp sliding window, (ii) primers’ matching allowed 2-nucleotide mismatching, and reads containing ambiguous bases were removed, and (iii) sequences with an overlap longer than 10 bp were merged according to their overlap sequence. The assembled sequences were assigned to operational taxonomic units (OTUs) at 97% similarity cutoff using UPARSE (Highly Accurate OTU Sequences from Microbial Amplicon Reads, version 7.1, http://drive5.com/uparse/, 30 September 2013) and chimeric sequences were identified and removed using UCHIME (Chimera Prediction for Amplicon Sequencing). OTUs were used for α-diversity (Coverage, Sobs, ACE, Chao, Shannon and Simpson) analysis. OTUs were taxonomically analyzed by the Ribosomal Database Project (RDP) Classifier algorithm (http://rdp.cme.msu.edu/, 30 September 2016). The rarefaction curves’ analysis with Mothur v.1.21.1 was performed to reflect the sequence depth. Principal coordinate analysis (PCoA) was performed using the weighted Unifrac distance with R Language.

### 2.5. Statistical Analysis

The data of growth performance, organ weight, blood parameters, and enzymatic activity were analyzed in a completely randomized design using the Proc Mixed procedure of SAS (SAS Inst. Inc., Cary, NC, USA). The model was Y_ij_ = μ + T_i_ + P_j_ + T_i_ × P_j_ + ε_ij_, where Y_ij_ was the dependent, continuous variable, μ was the overall mean, T_i_ was the fixed effect of diet treatment (i = basal diet, flaxseed oil, or flaxseed grain), P_j_ was the random effect of pen (j = 1, 2, 3, and 4), Ti × Pj was the fixed effect of the interaction between diet and pen, and ε_ij_ was the residual error. The mixed model included fixed effects of diet and random effects of pen. Pen was considered the experimental unit and the repeated measurement. The model used to analyze body weight gain considers 4 replicates (number of pens), each with 5 observations (number of goats): organ weight, blood parameters, and enzymatic activity consider 4 replicates (number of pens), each with 2 observations (number of goats), for each treatment. Specifically, the model used to study feed intake considers 4 replicates (number of pens), each with 1 observation. The results are presented as the mean values and standard error of the mean (SEM). Data means significance was declared at *p* ≤ 0.05 and tendencies were considered at 0.05 < *p* ≤ 0.10.

For evaluation of bacterial diversity indexes, the bacteria community structure at phylum and genus level, a one-way analysis of variance (ANOVA) and Duncan’s multiple range tests were carried out in SAS (SAS Inst. Inc., Cary, NC, USA). The results are presented as the mean values and SEM. Spearman correlation was used to correlate the growth performance, lipid deposition, blood lipid profiles, and blood lipid-related enzyme activity with the top 20 most relatively abundant bacterial genera through R Language (pheatmap package). Correlations with *p* ≤ 0.05 for the linear model were considered as significant.

## 3. Results

### 3.1. Growth Performance and Lipid Deposits

As indicated in Table 2 and Table 3, compared with the CON and HLS groups, the FBW, BWG, fat tissue weight, omental fat weight, and the percentage of fat tissue and omental fat to live body weight were significantly increased in the LNO group (*p* < 0.05), but the HLS group did not differ from the CON group. Compared with the HLS group, the weight of kidney fat and tail fat were remarkably increased in the LNO group (*p* = 0.041, *p* = 0.037), but the CON group did not differ from either LNO or HLS groups.

### 3.2. Blood Lipid Profiles

As shown in Table 4, compared with the HLS group, the contents of TG, CHO, and LDL-C were significantly increased in the CON and LNO groups (*p* = 0.001, *p* = 0.001, *p* = 0.001), the NEFA content showed the opposite result (*p* = 0.003), and the LNO group did not differ from the CON group. Compared with the CON and HLS treatments, the BHBA content significantly decreased in the LNO treatment (*p* = 0.002), and there was no difference between the CON and HLS treatments. The HDL-C content tended to be remarkably higher in the LNO and HLS groups than the CON group (*p* = 0.062), but the HLS group did not differ from the LNO group. The GLU content tended to be remarkably higher in the LNO treatment (*p* = 0.062) than the CON and HLS treatments, but the HLS treatment did not differ from the CON treatment.

### 3.3. The Quantity of Enzymes Related to Blood Lipid Metabolism

As indicated in Table 5, compared with the CON and HLS treatments, the ACC and MDH quantity remarkably increased in the LNO treatment (*p* = 0.015, *p* = 0.010), but there was no difference between the CON and HLS treatments. Compared with the CON and LNO treatments, the HSL quantity significantly increased in the HLS treatment (*p* = 0.002), but there was no difference between the CON and LNO treatments. The FAS quantity was significantly higher in the LNO treatment than the HLS treatment (*p* = 0.016), but the CON treatment did not differ from either LNO or HLS treatments.

### 3.4. Microbial Diversity of Ileum Digesta

#### 3.4.1. Sampling Depth

**Figure 1:**
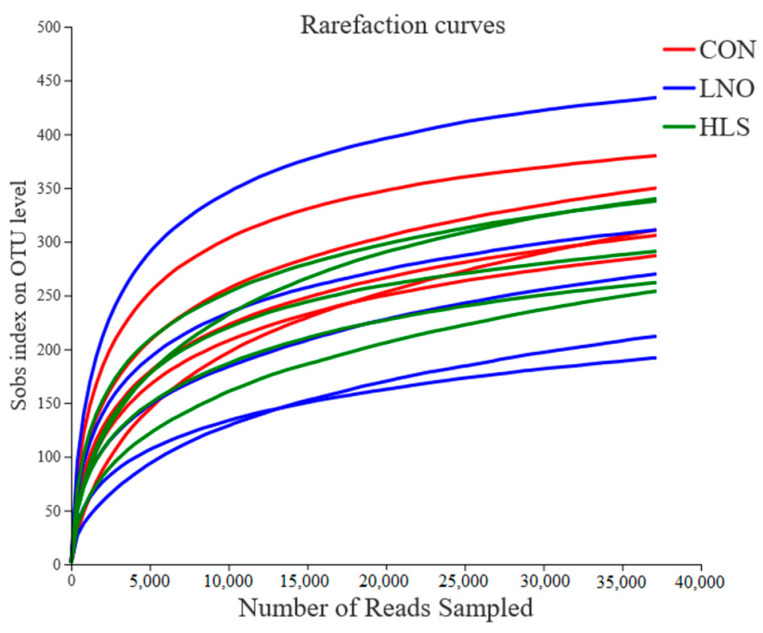
The OTU rarefaction curves of the ileum digesta bacterial communities. Curves were drawn using the least sequenced sample as the upper limit for the rarefactions. Each color represents one treatment: The red curves represent kids fed the basal diet (CON), the blue curves represent kids fed the basal diet supplemented with flaxseed oil (LNO), and the green curves represent kids fed the basal diet supplemented with heated flaxseed grain (HLS).

As shown in Table 6, after optimizing the original data, 271,857, 281,019, and 253,906 high-quality valid sequences were obtained in the CON, LNO, and HLS groups, respectively. On the basis of 97% species similarity, 563, 524, and 594 OTUs were separately obtained from samples in the CON, LNO, and HLS groups. Nearly 806,782 sequences were generated for ileum digesta bacteria, with an average of 54,371, 56,204, and 50,781 sequences for each sample in the CON, LNO, and HLS groups, respectively. Both the rarefaction curves (Figure 1) and the high coverage value (from 0.9985 to 0.9987, Table 4) showed that the sampling depth is enough to estimate bacterial diversity.

#### 3.4.2. Ileum Microbiota α- and β-Diversity

**Figure 2:**
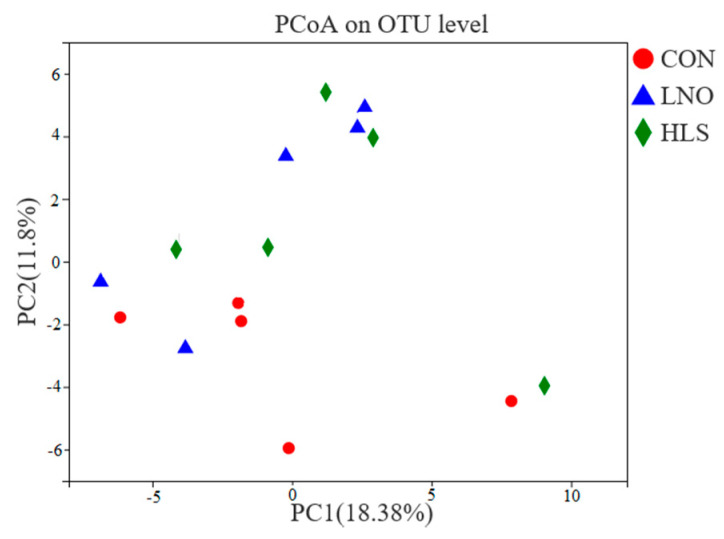
Principal coordinate analysis (PCoA) (using the weighted Unifrac similarity metric) of bacterial operational taxonomic units (OTUs) in the ileum digesta of goat kids. Each symbol represents one treatment: The solid red circle represents kids fed the basal diet (CON), the solid blue triangle represents kids fed the basal diet supplemented with flaxseed oil (LNO), and the solid green rhombus represents kids fed the basal diet supplemented with heated flaxseed grain (HLS).

As indicated in Table 7, compared with the LNO group, the Sobs (the number of OTUs), ACE (the ACE estimator) and Chao (the Chao estimator) index significantly increased in CON and HLS groups (*p* = 0.038, *p* = 0.001, *p* = 0.019), but there was no significant difference between CON and HLS groups. The principal coordinate analysis (PCoA) plots (Figure 2) demonstrate dissimilarities between the CON group, the LNO group, and the HLS group.

**Figure 3:**
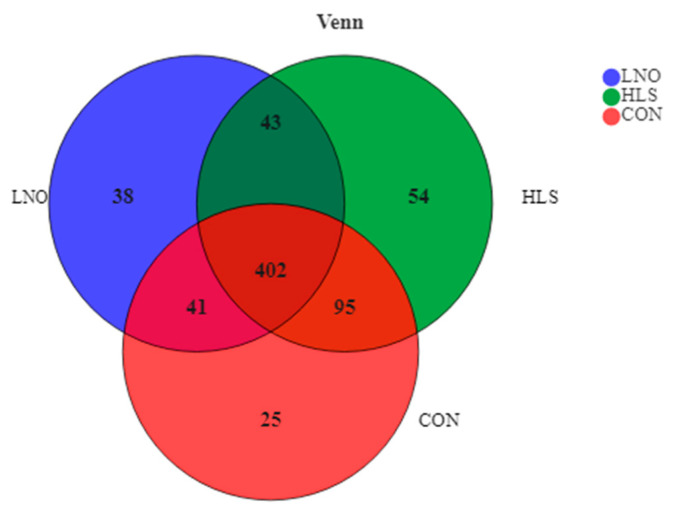
Venn plot of operational taxonomic units (OTUs) showing the percent of observations for each OTU (>0.5%) present in each dietary group (flaxseed oil, LNO, heated flaxseed grain, HLS, CON). Each color represents one treatment: The red represents kids fed the basal diet (CON), the blue represents kids fed the basal diet supplemented with flaxseed oil (LNO), and the green represents kids fed the basal diet supplemented with heated flaxseed grain (HLS).

A total of 698 OTUs were obtained from all samples, of which 402 exist in all groups defined as core OTUs (Figure 3). The core OTUs comprised approximately 57.6% of the total OTUs. In addition, 25, 38, and 54 OTUs were uniquely identified in groups CON, LNO, and HLS, respectively.

#### 3.4.3. Bacterial Community at Different Taxonomical Levels

Taxonomic assignment of OTUs identified 17 phyla in the ileum digesta of Albas cashmere goats. As shown in Table 8, a detailed overview of bacteria composition of ileum digesta in each group was illustrated at the phylum level. The Firmicutes is the most predominant phylum in the ileum digesta of the CON (90.80%), LNO (87.22%), and HLS (75.83%) groups, whereas Actinobacteria (CON: 4.96%, LNO: 8.92%, HLS: 20.06%) is secondary, they account for 95.76%, 96.14%, and 95.89% of total sequences, respectively. Compared with the CON and LNO groups, the relative abundance (RA) of Firmicutes, Proteobacteria, and Tenericutes reduced remarkably in the HLS group (*p* = 0.015, *p* = 0.042, *p* = 0.005), the Actinobacteria RA showed the opposite result (*p* = 0.001), and the LNO group did not differ from the CON group.

On the genus level, a total of 43 genera were identified from all samples. The 20 most abundant genera are listed in Table 9 for ileum samples. The RA of the top 20 bacterial genera were 73.08%, 84.88%, and 66.38% in the CON, LNO, and HLS groups, respectively. Compared with CON treatment, the RA of unclassified_f_Peptostreptococcaceae (Uncl. Pep.), Clostridium_sensu_stricto_1, and Lachnospiraceae_NK3A20_group significantly decreased in LNO and HLS treatments, and these genera were remarkably lower in the HLS group than in the LNO group (*p* < 0.0001), but the [Eubacterium]_coprostanoligenes_group ([E.] cop. group), Family_XIII_AD3011_group, and Aeriscardovia showed the opposite result (*p* < 0.0001). Compared with CON and LNO treatments, the RA of Intestinibacter remarkably decreased in HLS treatment (*p* = 0.001), but there was no difference between CON and LNO groups. Compared with CON treatment, the RA of Ruminococcus_2, Ruminococcaceae_UCG-014, and Christensenellaceae_R-7_group significantly increased in the LNO group, but these genera remarkably decreased in the HLS group (*p* < 0.0001). Compared with CON and HLS treatments, the Turicibacter RA remarkably increased in LNO treatment (*p* < 0.0001), the Streptococcus and [Ruminococcus]_gauvreauii_group showed the opposite result, and there was no difference between CON and HLS groups. Compared with CON treatment, the RA of Lactobacillus, Bifidobacterium, Olsenella, and Ureaplasma significantly increased in LNO and HLS treatments, and these genera were remarkably higher in LNO treatment than in HLS treatment (*p* < 0.0001). Compared with CON treatment, the RA of Acetitomaculum and Mycoplasma significantly decreased in LNO and HLS treatments, and these genera were remarkably lower in the LNO group than in the HLS group (*p* < 0.0001). The unclassified_f__Bifidobacteriaceae RA tended to be remarkably lower in the HLS group than in the CON group (*p* = 0.064), but the LNO group did not differ from the CON and HLS groups.

#### 3.4.4. Spearman Correlation Analysis

Spearman correlation analysis was conducted between the abundance of the top 20 bacterial genera and growth performance, fat tissue, blood lipid profiles, and the enzymes related to blood lipid metabolism, as indicated in Table 10, Table 11 and Table 12. The threshold |R| > 0.5 and *p* ≤ 0.05 is considered as a significant Spearman correlation. The [Ruminococcus]_gauvreauii_group was significantly positively correlated with BHBA and NEFA, but negatively associated with BWG, Omental Fat, and GLU. The Streptococcus was positively correlated with BHBA and NEFA, but negatively associated with FBW, BWG, Omental Fat, Kidney Fat, GLU, ACC, FAS, and MDH. The Turicibacter was positively correlated with FBW, BWG, Omental Fat, CHO, ACC, and FAS, but negatively associated with BHBA and NEFA. The Acetitomaculum and Mycoplasma were negatively associated with BWG, Omental Fat, GLU, HDL-C, and ACC. The Bifidobacterium was positively correlated with BWG, Omental Fat, Mesenteric Fat, GLU, HDL-C, and ACC, but negatively associated with BHBA and NEFA. The Christensenellaceae_R-7_group was positively correlated with BWG, Omental Fat, CHO, LDL-C, and ACC, but negatively associated with BHBA, NEFA, and HSL. The Olsenella was positively correlated with BWG and GLU. The Ruminococcaceae_UCG-014 was positively correlated with BWG, Omental Fat, CHO, LDL-C, ACC, and MDH, but negatively associated with BHBA, NEFA, and HSL. The Ruminococcus_2 was positively correlated with FBW, BWG, Omental Fat, GLU, CHO, LDL-C ACC, FAS, and MDH, but negatively associated with BHBA, NEFA, and HSL. The Ureaplasma was positively correlated with BWG, Omental Fat, and GLU, but negatively associated with NEFA. The Lactobacillus was positively correlated with GLU. The Lachnospiraceae_NK3A20_group was positively correlated with TG and LDL-C, but negatively associated with HSL. The Intestinibacter was positively correlated with MDH, but negatively associated with BHBA and NEFA. The Family_XIII_AD3011_group was positively correlated with HSL, but negatively associated with LDL-C. The Clostridium_sensu_stricto_1 was positively correlated with LDL-C, but negatively associated with HSL. The unclassified_f__Bifidobacteriaceae was negatively associated with HDL-C. The Aeriscardovia was positively correlated with HSL, but negatively associated with LDL-C. The Uncl. Pep. Was negatively associated with HSL and LPL, but the [E.] cop. group was positively correlated with them.

## 4. Discussion

Our previous study has shown that, compared with the addition of flaxseed oil, flaxseed could protect ALA from the hydrogenation in rumen, increase the ALA content in blood, and then increase the ALA accumulation in muscle of goats, which is beneficial to human health [10,17]. However, the comparison effects of flaxseed oil and flaxseed grain on lipid deposition and ileum microbiota profiles have not been investigated. The present study indicated that flaxseed is more effective than flaxseed oil in reducing lipid deposition, which may work by modulating the ileal microbiota composition.

Candyrine et al. reported that 4% oil supplementation to diet significantly enhanced the growth performance in fattening sheep and goats [22]. In the current study, compared with the CON group, the dietary flaxseed oil supplementation significantly increased the FBW and BWG, which agrees with the above report. Compared with the CON treatment, flaxseed oil significantly promoted the FBW, BWG, and lipid accumulation in goats, but the dietary flaxseed supplementation had no effect on the growth performance of goats. However, compared with the LNO group, the dietary flaxseed supplementation reduced the adipose tissue weight, indicating that flaxseed efficiently reduced the lipid deposition of goats. Blood biochemical parameters can reflect the physiological and metabolic state of animals, which is closely related to nutritional status. The present results showed that the dietary flaxseed grain supplementation significantly decreased the content of TG, CHO, and LDL-C in serum, indicating that flaxseed could reduce the blood lipid deposition, which is consistent with the results of changes in adipose tissue weight. In agreement with these findings, the majority of previous studies showed that flaxseed consumption reduced serum content of CHO [23] and LDL-C [24] levels. In the current study, compared with the CON and HLS groups, the flaxseed oil had a tendency to increase the serum GLU content and significantly reduced the serum BHBA content, which suggested that the addition of flaxseed oil to the diet reduced lipid mobilization, resulting in an increase of lipid deposition. Compared with the LNO group, flaxseed significantly increased the serum BHBA and NEFA content, which indicated that cashmere goats would mobilize lipid to meet growth needs, so they produced a large amount of BHBA and NEFA. ACC and FAS are the key enzymes involved in de novo FA synthesis and play a key role in animal lipid synthesis [10]. MDH is closely involved in the trans hydrogenation during the conversion of carbohydrates to fatty acids [25]. HSL is the key enzyme and rate-limiting enzyme that initially mobilized lipolysis, it gradually hydrolyzes the fat stored in fat cells into free FA and glycerin and releases them into the blood [26]. In the current study, compared with the LNO treatment, the flaxseed grain significantly decreased the quantity of FAS, ACC, and MDH, but remarkably promoted the HSL quantity. These results suggested that adding flaxseed grain to the diet could reduce the quantity of enzymes related to lipid de novo synthesis and increase the quantity of enzymes related to lipolysis, thereby reducing the blood TG content and TG accumulation in the adipose tissue, which further explained the above-mentioned change of growth performance and lipid deposition. Liu et al. reported that the diets containing unsaturated fatty acids (UFAs) could reduce serum CHO, TG, and LDL-C content, but increase serum HDL-C content [27]. Egert et al. observed that enrichment of the diet with EPA or DHA decreased fasting serum TG concentration by 15% and 31% in humans, suggesting that DHA is more effective in reducing TG levels in the blood [28]. Morin et al. reported a lower serum CHO and LDL-C levels of rats in the DHA monoglycerides treatment than the high-fat/high-carbohydrate diet [29]. In our previous study, the flaxseed grain group had a higher plasma concentration of DHA than the control and supplemented flaxseed oil groups [10], but in the present study, had a lower serum TG, CHO, and LDL-C content, hinting that flaxseed grain is more efficient to goats in reducing the blood TG, CHO, and LDL-C level because of the decreased plasma DHA. Sawada et al. reported that the EPA significantly improved the HDL-C concentration in blood of humans [30]. HDL-C removes cholesterol from the bloodstream and carries it back to the liver for recycling [31], which is beneficial to the health of livestock. In the present study, the diet supplemented with flaxseed oil and flaxseed grain had a tendency to increase the serum HDL-C concentration compared to the CON group, which probably related to the increase of plasma EPA concentration [10]. Some researches indicated that the lipid-lowering effect of flaxseed is owing to its high content of lignans and soluble fibers [32,33]. Lignans are one of the vital groups of phytoestrogens, which inhibit acyl-coenzyme A activity: CHO acyltransferase involved in the formation of cholesteryl ester in tissues [34,35]. Soluble fibers can reduce intestinal absorption of dietary CHO and increase the production of bile acid [33]. In conclusion, supplementation of flaxseed significantly alleviated the lipid accumulation in goats compared to the addition of flaxseed oil, indicating that flaxseed (enriched ALA) may play an important role in regulating lipid metabolism, and therefore it is beneficial to the goats.

In recent years, the gut microbiota has been shown to affect lipid levels and lipid metabolism in blood. In the present study, the dietary flaxseed supplementation significantly reduced the relative abundance of *Firmicutes*, *Proteobacteria*, and *Tenericutes*, while it remarkably increased the *Actinobacteria* relative abundance. *Firmicutes* is known as obesity-related bacterial phylum, which accelerates degradation of food components to supply energy for the host [36]. A high abundance of *Firmicutes* goes with a low abundance of *Bacteroidetes*, which leads to accelerate energy harvest from food and promote energy storage in adipose tissue of the host [37] and would further suppress the fasting-induced adipose factor (FIAF) produced. Meanwhile, a higher TG was stored in adipose tissue and lower satiety hormones were released by the suppression of FIAF [38]. In the current study, the dietary flaxseed significantly decreased the *Firmicutes* relative abundance compared to the CON and LNO groups, so we speculated that adding flaxseed to the diet promoted the production of FIAF by reducing the *Firmicutes* relative abundance, thereby releasing more satiety hormones, reducing the absorption of energy from the diet through ileum, which further explained the decrease in blood TG and fat tissue weight. *Proteobacteria* is a major phylum of Gram-negative bacteria with a wide range of pathogenic microorganisms, and a high ratio of *Proteobacteria* to *Firmicutes + Bacteroidetes* was confirmed as a good indicator for rumen dysbiosis [39]. The addition of flaxseed decreased the Proteobacteria relative abundance in ileum of goats, which may be caused by competitive relationships between the gut microbiota and pathogenic bacteria in the intestine [40], indicating that the flaxseed is beneficial to the colonization of probiotic in the goats’ ileum. *Actinobacteria* is the dominant bacterial in most mammals [41]. In the long-term co-evolution process of animal intestinal *Actinobacteria* and the host, by producing a variety of bioactive substances, such as antibiotics, immunosuppressants, vitamins, and enzymes, they participate in the metabolism of the host and maintain the intestinal microecological balance of the host and a series of important physiological activities [42]. Claus et al. reported that the *Actinobacteria* was negatively associated with serum GLU level in the microbial colonization process [43]. The current study observed that, compared to added flaxseed oil, dietary flaxseed supplementation increased the *Actinobacteria* relative abundance and had a tendency to reduce the serum GLU content, which is similar to the above-mentioned reports.

*[E.] cop.* is a CHO-reducing bacterium, and on the one hand, converting CHO to coprostanol could decrease the CHO absorption [44]. On the other hand, the bacterium might interrupt the enterohepatic circulation of billary CHO so that the liver would partition more CHO into the bile and less CHO into the blood [45]. In the current study, *[E.] cop. group* (16.92%), *Aeriscardovia* (14.95%), and *Uncl. Pep.* (13.13%) were the 3 most abundant genera in the goats’ ileum of the HLS group, which showed that the flaxseed significantly increased the *[E.] cop. group* relative abundance and remarkably decreased the blood CHO content, and that the *[E.] cop. group* was positively correlated with the content of HSL and LPL, illustrating that the flaxseed can reduce the absorption of CHO in ileum and increase the degradation of lipid by increasing the relative abundance of *[E.] cop. group*. Whon et al. reported that the castrated male cattle harbor distinct ileum microbiota which were dominated by the family *Peptostreptococcaceae*, with increased extra- and intra-muscular fat storage [46]. A previous study revealed that the *Uncl. Pep.* might play an important role in feed digestion [47]. In the current study, *Uncl. Pep.* (31.22%), *Intestinibacter* (12.01%), and *Ruminococcus_2* (10.61%) were the 3 most abundant genera in the goats’ ileum of the LNO group, *Uncl. Pep.* (35.14%) and *Intestinibacter* (13.73%) were the 2 most abundant genera in the goats’ ileum of the CON group, the relative abundance of *Uncl. Pep.* and *Intestinibacter* are significantly higher in the CON and LNO groups than in the HLS group, and the blood TG content showed a consistent trend of change. Meanwhile, the current results showed that the *Uncl. Pep.* was negatively associated with the quantity of HSL and LPL, and the *Intestinibacter* was negatively associated with the content of BHBA and NEFA. These results hinted that the increased absorption of nutrients in ileum and the reduced mobilization of body fat in goats of CON and LNO groups was due to increasing the relative abundance of *Uncl. Pep.* and *Intestinibacter*. Jiang et al. reported that the increase of *Ruminococcus* relative abundance can increase the digestibility of DM and NDF in vivo and the production performance of cows [48]. The dietary supplementation of flaxseed oil significantly elevated the *Ruminococcus_2* relative abundance and increased the FBW and BWG of goats, and the *Ruminococcus_2* genus was positively associated with FBW, BWG, Omental Fat, GLU, CHO, LDL-C, ACC, and MDH, but negatively correlated with BHBA, NEFA, and HSL. Therefore, these results hinted that the flaxseed oil can promote the absorption of nutrients and reduce lipid mobilization, leading to much more GLU and TG flowed into blood, more lipid deposition in adipose tissue, and increasing the FBW of goats by increasing the *Ruminococcus_2* relative abundance.

The present study investigated the shift among blood lipid profiles in cashmere goats in response to dietary supplementation of flaxseed or flaxseed oil, which probably related to the change of ileal microbial composition, but the mechanism is unclear. Therefore, the ileal metabolites of goats fed a diet containing flaxseed or flaxseed oil needed to be measured to further elucidate the mechanism in the future. The *[E.] cop.* is the most relatively abundant genus in the ileum of HLS goats and the *Ruminococcus_2* is the third top genus in the ileum of LNO goats, and they are strongly associated with host lipid metabolism, so the function of *[E.] cop.* and *Ruminococcus_2* on lipid metabolism needed to be verified in vitro. In addition, further study is needed to test other intestinal microbes (duodenum, jejunum, cecum, and colon), to better interpret the microbiota compositions and the shift of microbiota in response to dietary supplementation of flaxseed or flaxseed oil.

## 5. Conclusions

In conclusion, the flaxseed grain is more efficient than flaxseed oil in ameliorating the blood lipid profiles and it is a potential product in decreasing the lipid deposition of cashmere goats. The blood lipid profiles in cashmere goats in response to dietary supplementation of flaxseed or flaxseed oil are probably related to the change of the ileal microbiota composition.

## Figures and Tables

**Table 1:** The composition and nutrient levels of CON, LNO, and HLS group (dry-matter basis, DM basis).

Ingredients	1 to 30 Days			31 to 60 Days			61 to 90 Days		
	CON	LNO	HSL	CON	LNO	HSL	CON	LNO	HSL
Alfalfa	25.00	25.00	25.00	15.00	15.00	15.00	12.50	12.50	12.50
Corn stalk	5.00	5.00	5.00	20.00	20.00	20.00	25.00	25.00	25.00
Oat	20.00	20.00	20.00	15.00	15.00	15.00	12.50	12.50	12.50
Corn	28.41	23.37	23.17	30.80	30.40	29.90	31.30	29.90	29.40
Soybean meal 46%	11.70	10.50	11.50	9.50	11.40	11.90	8.00	10.40	10.90
Distillers dried grains with solubles, DDGS	3.00	7.24	7.74	4.00	0.50	0.50	4.00	0.50	0.50
Flaxseed meal	4.80	4.80	0.00	3.50	3.50	0.00	4.50	4.50	0.00
Flaxseed	0.00	0.00	5.50	0.00	0.00	5.50	0.00	0.00	7.00
Flaxseed oil	0.00	2.00	0.00	0.00	2.00	0.00	0.00	2.50	0.00
Premix ^(1)^	0.50	0.50	0.50	0.50	0.50	0.50	0.50	0.50	0.50
Limestone	0.2	0.2	0.2	0.2	0.2	0.2	0.2	0.2	0.2
CaHPO_4_	0.2	0.2	0.2	0.2	0.2	0.2	0.2	0.2	0.2
NaCl	0.54	0.54	0.54	0.50	0.50	0.50	0.50	0.50	0.50
NaHCO_3_	0.35	0.35	0.35	0.80	0.80	0.80	0.80	0.80	0.80
MgO	0.30	0.30	0.30	0.00	0.00	0.00	0.00	0.00	0.00
Total	100.00	100.00	100.00	100.00	100.00	100.00	100.00	100.00	100.00
Nutrient levels									
Digestible energy, DE MJ/Kg DM ^(2)^	12.83	13.09	13.06	12.87	13.00	12.96	12.74	13.09	13.05
Dry Matter, DM/%	88.02	88.36	88.24	89.14	89.36	89.32	87.09	87.02	87.03
Crude protein, CP g/kg DM	188.73	188.13	188.20	162.84	158.71	159.69	153.52	151.34	151.85
Ether extract, EE g/kg DM	29.12	53.97	53.97	28.99	45.84	46.14	26.85	48.99	49.92
Neutral Detergent Fiber, NDF g/kg DM	425.31	431.18	441.6	448.6	427.41	439.12	457.42	436.06	450.68
Acid Detergent Fiber, ADF g/kg DM	232.2	237.71	243.3	242.59	235.23	248.4	247.69	240.32	256.96
Calcium, Ca g/kg DM	11.25	11.11	11.00	10.48	10.89	10.78	10.26	10.67	10.56
Phosphorus, P g/kg DM	4.65	4.67	4.78	4.50	4.44	4.56	4.31	4.22	4.33

^(1)^ Per kilogram of premix provided the following: iron (Fe) 4 g, copper (Cu) 0.8 g, zinc (Zn) 5 g, manganese (Mn) 3 g, iodine (I) 30 mg, selenium (Se) 30 mg, cobalt (Co) 25 mg, vitamin (VA) 600,000 IU (International Unit), vitamin D (VD3) 250,000 IU, vitamin E (VE) 1250 IU, vitamin K (VK3) 180 mg, vitamin B1 (VB1) 35 mg, vitamin B2 (VB2) 850 mg, vitamin B6 (VB6) 90 mg, nicotinic acid 2200 mg, D-pantothenic acid 1700 mg, vitamin B12 (VB12) 3 mg, biotin 14 mg, folic acid 150 mg. ^(2)^ Digestible energy was calculated based on the ingredients of the diet and their digestible energy content, not based on the actual dry matter intake. CON: basal diet; LNO: basal diet added with flaxseed oil; HLS: basal diet added with heated flaxseed grain.

**Table 2:** The effect of dietary flaxseed oil or flaxseed supplementation on the growth performance in Albas cashmere goats.

Items	CON	LNO	HLS	SEM	*p*-Value
Feed Intake (kg/day/per)	0.72	0.79	0.71	0.041	0.379
Initial Body Weight/kg	18.65	18.54	18.51	0.245	0.915
Final Body Weight/kg	26.74 ^B^	28.14 ^A^	26.51 ^B^	0.370	0.006
Total Body Weight Gain/kg	8.03 ^B^	9.80 ^A^	7.95 ^B^	0.382	0.0001

^A, B^ Means within the same row not followed by the same letters are significantly different at *p* ≤ 0.05, whereas the differences were considered to be a statistical trend when 0.05 < *p* ≤ 0.10. CON: basal diet; LNO: basal diet added with flaxseed oil; HLS: basal diet added with heated flaxseed grain. SEM: standard error of the mean.

**Table 3:** The effect of dietary flaxseed oil or flaxseed supplementation on the lipid deposits in Albas cashmere goats.

Items	CON	LNO	HLS	SEM	*p*-Value
Fat Tissue Weight/g					
Kidney Fat	129.93 ^AB^	153.91 ^A^	120.63 ^B^	8.352	0.041
Omental Fat	729.31 ^B^	1053.55 ^A^	696.62 ^B^	12.006	<0.0001
Mesenteric Fat	487.49	547.97	417.84	50.527	0.231
Tail Fat	12.39 ^AB^	14.70 ^A^	11.16 ^B^	0.853	0.037
Fat Tissue	1473.59 ^B^	1843.90 ^A^	1249.60 ^B^	107.680	0.007
Percentage of Live Body Weight/%					
Kidney Fat	0.50	0.54	0.47	0.030	0.294
Omental Fat	2.77 ^B^	4.10 ^A^	2.83 ^B^	0.096	<0.0001
Mesenteric Fat	1.88	1.94	1.79	0.153	0.797
Tail Fat	0.05	0.05	0.04	0.004	0.771
Fat Tissue	5.19 ^B^	6.45 ^A^	5.07 ^B^	0.151	<0.0001

^A, B^ Means within the same row not followed by the same letters are significantly different at *p* ≤ 0.05, whereas the differences were considered to be a statistical trend when 0.05 < *p* ≤ 0.10. CON: basal diet; LNO: basal diet added with flaxseed oil; HLS: basal diet added with heated flaxseed grain. SEM: standard error of the mean.

**Table 4:** The effect of dietary flaxseed oil or flaxseed supplementation on the blood lipid profiles in Albas cashmere goats.

Items	CON	LNO	HLS	SEM	*p*-Value
TG (mmol/L)	0.63 ^A^	0.64 ^A^	0.48 ^B^	0.036	0.001
CHO (mmol/L)	1.87 ^A^	2.11 ^A^	1.57 ^B^	0.100	0.001
LDL-C (mmol/L)	0.54 ^A^	0.57 ^A^	0.40 ^B^	0.026	0.001
HDL-C (mmol/L)	0.82	0.86	0.87	0.015	0.062
GLU (mmol/L)	3.64	3.88	3.72	0.093	0.062
NEFA (nmol/L)	0.23 ^B^	0.16 ^B^	0.36 ^A^	0.035	0.003
BHBA (mmol/L)	0.20 ^A^	0.14 ^B^	0.23 ^A^	0.013	0.002

^A, B^ Means within the same row not followed by the same letters are significantly different at *p* ≤ 0.05, whereas the differences were considered to be a statistical trend when 0.05 < *p* ≤ 0.10. CON: basal diet; LNO: basal diet added with flaxseed oil; HLS: basal diet added with heated flaxseed grain. SEM: standard error of the mean. TG: triglyceride; CHO: cholesterol; LDL-C: low-density lipoprotein cholesterol; HDL-C: high-density lipoprotein cholesterol; GLU: glucose; NEFA: non-esterified fatty acid; BHBA: β-hydroxybutyric acid.

**Table 5:** The effect of dietary flaxseed oil or flaxseed supplementation on the enzymes related to lipid metabolism in blood of Albas cashmere goats.

Items	CON	LNO	HLS	SEM	*p*-Value
FAS (ng/mL)	26.07 ^AB^	31.07 ^A^	22.57 ^B^	1.748	0.016
ACC (ng/mL)	82.26 ^B^	100.38 ^A^	81.15 ^B^	4.365	0.015
MDH (ng/mL)	18.24 ^B^	19.24 ^A^	17.58 ^B^	0.470	0.010
LPL (ng/mL)	340.24	334.40	364.28	12.551	0.134
HSL (ng/mL)	7.21 ^B^	7.34 ^B^	9.55 ^A^	0.393	0.002
LPS (ng/mL)	9.80	10.00	8.87	0.424	0.176

^A, B^ Means within the same row not followed by the same letters are significantly different at *p* ≤ 0.05, whereas the differences were considered to be a statistical trend when 0.05 < *p* ≤ 0.10. CON: basal diet; LNO: basal diet added with flaxseed oil; HLS: basal diet added with heated flaxseed grain. SEM: standard error of the mean. FAS: fatty acid synthetase; ACC: acetyl-coa carboxylase; MDH: malic dehydrogenase; LPL: lipoprotein lipase; HSL: hormone-sensitive lipase; LPS: lipase.

**Table 6:** Sequence data of bacteria in ileum digesta.

Group	Optimized Sequences	OTUs	Average Optimized Sequences of Sample	Average Length of Optimized Sequence
CON	271,857	563	54,371	429
LNO	281,019	524	56,204	431
HLS	253,906	594	50,781	433

CON: basal diet; LNO: basal diet added with flaxseed oil; HLS: basal diet added with heated flaxseed grain. OTUs: operational taxonomic units.

**Table 7:** The α-diversity indexes of bacteria in ileum digesta.

Index	CON	LNO	HLS	SEM	*p*-Value
Coverage	0.9985	0.9987	0.9985	0.0002	0.722
Sobs	325.25 ^A^	253.00 ^B^	354.50 ^A^	12.867	0.038
ACE	418.86 ^A^	356.34 ^B^	404.77 ^A^	3.713	0.001
Chao	407.55 ^A^	354.18 ^B^	408.35 ^A^	5.753	0.019
Shannon	2.69	2.56	2.64	0.194	0.931
Simpson	0.16	0.14	0.17	0.025	0.748

^A, B^ Means within the same row not followed by the same letters are significantly different at *p* ≤ 0.05, whereas the differences were considered to be a statistical trend when 0.05 < *p* ≤ 0.10. CON: basal diet; LNO: basal diet added with flaxseed oil; HLS: basal diet added with heated flaxseed grain. SEM: standard error of the mean. Sobs: the number of OTUs; ACE: the ACE estimator; Chao: the Chao estimator.

**Table 8:** The effect of dietary flaxseed oil or flaxseed supplementation on the relative abundance of bacteria in ileum digesta—Phylum level (%).

Phylum	CON	LNO	HLS	SEM	*p*-Value
Firmicutes	90.80 ^A^	87.22 ^A^	75.83 ^B^	1.826	0.015
Actinobacteria	4.96 ^B^	8.92 ^B^	20.06 ^A^	1.449	0.001
Proteobacteria	0.79 ^A^	0.71 ^A^	0.37 ^B^	0.106	0.042
Tenericutes	1.25 ^A^	1.03 ^A^	0.62 ^B^	0.102	0.005
Bacteroidetes	1.48	1.49	2.78	0.641	0.292
others	0.70	0.67	0.42	0.237	0.776

^A, B^ Means within the same row not followed by the same letters are significantly different at *p* ≤ 0.05, whereas the differences were considered to be a statistical trend when 0.05 < *p* ≤ 0.10. CON: basal diet; LNO: basal diet added with flaxseed oil; HLS: basal diet added with heated flaxseed grain. SEM: standard error of the mean.

**Table 9:** The effect of dietary flaxseed oil or heated flaxseed supplementation on the relative abundance of the top 20 bacteria in ileum digesta—Genus level (%).

Family	Genus	CON	LNO	HLS	SEM	*p*-Value
Phylum—Firmicutes						
Peptostreptococcaceae	g__unclassified_f__Peptostreptococcaceae	35.14 ^A^	31.22 B	13.13 ^C^	0.610	<0.0001
Peptostreptococcaceae	g__Intestinibacter	13.73 ^A^	12.01 A	9.32 ^B^	0.615	0.001
Clostridiaceae_1	g__Clostridium_sensu_stricto_1	4.13 ^A^	2.03 B	1.83 ^C^	0.055	<0.0001
Ruminococcaceae	g__Ruminococcus_2	3.32 ^B^	10.61 A	0.42 ^C^	0.561	<0.0001
Lachnospiraceae	g__Lachnospiraceae_NK3A20_group	2.58 ^A^	1.94 B	1.46 ^C^	0.104	<0.0001
Ruminococcaceae	g__[Eubacterium]_coprostanoligenes_group	2.54 ^C^	4.89 ^B^	16.92 ^A^	0.598	<0.0001
Erysipelotrichaceae	g__Turicibacter	1.63 ^B^	2.84 ^A^	1.58 ^B^	0.095	<0.0001
Ruminococcaceae	g__Ruminococcaceae_UCG-014	1.51 ^B^	4.10 ^A^	0.12 ^C^	0.204	<0.0001
Lachnospiraceae	g__Acetitomaculum	1.43 ^A^	0.32 ^C^	0.66 ^B^	0.092	<0.0001
Christensenellaceae	g__Christensenellaceae_R-7_group	1.34 ^B^	4.72 ^A^	0.61 ^C^	0.052	<0.0001
Family_XIII	g__Family_XIII_AD3011_group	0.81 ^C^	1.07 ^B^	1.59 ^A^	0.078	<0.0001
Streptococcaceae	g__Streptococcus	0.74 ^A^	0.36 ^B^	0.74 ^A^	0.036	<0.0001
Lachnospiraceae	g__[Ruminococcus]_gauvreauii_group	0.61 ^A^	0.13 ^B^	0.61 ^A^	0.058	<0.0001
Lactobacillaceae	g__Lactobacillus	0.05 ^C^	0.88 ^A^	0.22 ^B^	0.036	<0.0001
Phylum—Actinobacteria						
Bifidobacteriaceae	g__Aeriscardovia	2.08 ^C^	3.13 ^B^	14.95 ^A^	0.325	<0.0001
Bifidobacteriaceae	g__Bifidobacterium	0.27 ^C^	0.41 ^A^	0.32 ^B^	0.012	<0.0001
Bifidobacteriaceae	g__unclassified_f__Bifidobacteriaceae	0.26	0.23	0.17	0.025	0.064
Coriobacteriaceae	g__Olsenella	0.16 ^C^	3.73 ^A^	1.26 ^B^	0.028	<0.0001
Phylum–Tenericutes						
Mycoplasmataceae	g__Mycoplasma	0.73 ^A^	0.21 ^C^	0.44 ^B^	0.036	<0.0001
Mycoplasmataceae;	g__Ureaplasma	0.02 ^C^	0.05 ^A^	0.03 ^B^	0.002	<0.0001

^A–C^ Means within the same row not followed by the same letters are significantly different at *p* ≤ 0.05, whereas the differences were considered to be a statistical trend when 0.05 < *p* ≤ 0.10. CON: basal diet; LNO: basal diet added with flaxseed oil; HLS: basal diet added with heated flaxseed grain. SEM: standard error of the mean.

**Table 10:** Growth performance and lipid deposition showing a Spearman’s correlation with ileal bacterial community.

Items	FBW		BWG		Omental Fat		Kidney Fat		Mesenteric Fat	
	R ^(1)^	*p*-Value ^(2)^	R	*p*-Value	R	*p*-Value	R	*p*-Value	R	*p*-Value
g__[Ruminococcus]_gauvreauii_group			−0.52	0.04	−0.72	0.002				
g__Streptococcus	−0.55	0.04	−0.64	0.01	−0.82	0.0002	−0.52	0.05		
g__Turicibacter	0.58	0.02	0.73	0.002	0.74	0.001				
g__Acetitomaculum			−0.64	0.01	−0.59	0.02				
g__Bifidobacterium			0.77	0.001	0.63	0.01			0.62	0.01
g__Christensenellaceae_R-7_group			0.66	0.01	0.81	0.0002				
g__Mycoplasma			−0.61	0.02	−0.52	0.05				
g__Olsenella			0.60	0.02						
g__Ruminococcaceae_UCG-014			0.54	0.04	0.83	0.0001				
g__Ruminococcus_2	0.53	0.04	0.62	0.01	0.88	<0.0001				
g__Ureaplasma			0.58	0.02	0.54	0.04				

^(1)^ Spearman correlation coefficient (R) represents the degree of association between ileum bacterial community and growth performance, lipid deposition. ^(2)^ Correlations with *p* ≤ 0.05 for the linear model were considered as significant. FBW: final body weight; BWG: total body weight gain.

**Table 11:** Blood lipid profiles showing a Spearman’s correlation with ileal bacterial community.

Items	GLU		BHBA		NEFA		TG		CHO		LDL-C		HDL-C	
	R ^(1)^	*p*-Value ^(2)^	R	*p*-Value	R	*p*-Value	R	*p*-Value	R	*p*-Value	R	*p*-Value	R	*p*-Value
g__[Ruminococcus]_gauvreauii_group	−0.57	0.03	0.64	0.01	0.71	0.003								
g__Streptococcus	−0.57	0.03	0.78	0.0006	0.71	0.003								
g__Turicibacter			−0.70	0.004	−0.76	0.001			0.56	0.03				
g__Acetitomaculum	−0.71	0.003											−0.55	0.03
g__Bifidobacterium	0.55	0.03	−0.54	0.04	−0.62	0.01							0.60	0.02
g__Christensenellaceae_R-7_group			−0.88	<0.0001	−0.94	<0.0001			0.69	0.005	0.73	0.002		
g__Mycoplasma	−0.56	0.03											−0.56	0.03
g__Olsenella	0.59	0.02												
g__Ruminococcaceae_UCG-014			−0.92	<0.0001	−0.91	<0.0001			0.62	0.01	0.58	0.02		
g__Ruminococcus_2	0.52	0.05	−0.92	<0.0001	−0.88	<0.0001			0.63	0.01	0.52	0.05		
g__Ureaplasma	0.55	0.03			−0.55	0.03								
g__Lactobacillus	0.60	0.02												
g__Lachnospiraceae_NK3A20_group							0.53	0.04			0.64	0.01		
g__Intestinibacter			−0.63	0.01	−0.55	0.03								
g__Family_XIII_AD3011_group											−0.62	0.01		
g__Clostridium_sensu_stricto_1											0.58	0.02		
g__unclassified_f__Bifidobacteriaceae													−0.65	0.01
g__Aeriscardovia											−0.55	0.03		

^(1)^ Spearman correlation coefficient (R) represents the degree of association between ileum bacterial community and blood lipid profiles. ^(2)^ Correlations with *p ≤* 0.05 for the linear model were considered as significant. TG: triglyceride; CHO: cholesterol; LDL-C: low-density lipoprotein cholesterol; HDL-C: high-density lipoprotein cholesterol; GLU: glucose; NEFA: non-esterified fatty acid; BHBA: β-hydroxybutyric acid.

**Table 12:** The enzymes related to blood lipid metabolism showing a Spearman’s correlation with ileal bacterial community.

Items	ACC		FAS		HSL		LPL		MDH	
	R ^(1)^	*p*-Value ^(2)^	R	*p*-Value	R	*p*-Value	R	*p*-Value	R	*p*-Value
g__Streptococcus	−0.56	0.03	−0.64	0.01					−0.78	0.001
g__Turicibacter	0.71	0.003	0.52	0.04						
g__Acetitomaculum	−0.60	0.02								
g__Bifidobacterium	0.60	0.02								
g__Christensenellaceae_R-7_group	0.59	0.02			−0.70	0.004				
g__Mycoplasma	−0.52	0.05								
g__Ruminococcaceae_UCG-014	0.57	0.03			−0.53	0.04			0.57	0.03
g__Ruminococcus_2	0.57	0.03	0.54	0.04	−0.52	0.05			0.67	0.01
g__Lachnospiraceae_NK3A20_group					−0.78	0.001				
g__Intestinibacter									0.51	0.05
g__Family_XIII_AD3011_group					0.78	0.001				
g__Clostridium_sensu_stricto_1					−0.77	0.001				
g__unclassified_f__Peptostreptococcaceae					−0.67	0.01	−0.56	0.03		
g__[Eubacterium]_coprostanoligenes_group					0.71	0.003	0.56	0.03		
g__Aeriscardovia					0.75	0.001				

^(1)^ Spearman correlation coefficient (R) represents the degree of association between ileum bacterial community and enzymes related to blood lipid metabolism. ^(2)^ Correlations with *p* ≤ 0.05 for the linear model were considered as significant. FAS: fatty acid synthetase; ACC: acetyl-coa carboxylase; MDH: malic dehydrogenase; LPL: lipo-protein lipase; HSL: hormone-sensitive lipase.

## Data Availability

The raw data presented in the current study are available on reasonable request from the corresponding author.
